# Pattern and Trend of Substance Abuse in Eastern Rural Iran: A Household Survey in a Rural Community

**DOI:** 10.1155/2013/297378

**Published:** 2013-12-24

**Authors:** Hasan Ziaaddini, Tayebeh Ziaaddini, Nouzar Nakhaee

**Affiliations:** ^1^Research Center for Health Services Management, Institute of Futures Studies in Health, Kerman University of Medical Sciences, Kerman, Iran; ^2^Research Center for Social Determinants of Health, Institute of Futures Studies in Health, Kerman University of Medical Sciences, Kerman, Iran; ^3^Neuroscience Research Center, Institute of Neuropharmacology, Kerman University of Medical Sciences, P.O. Box 76175-113, Kerman, Iran

## Abstract

*Introduction and Aim*. Substance abuse imposes hazards on human health in all biopsychosocial aspects. Limited studies exist on epidemiology of substance abuse and its trend in rural areas. The present study aimed to compare substance abuse in one of the rural areas of southeast Iran, in a 12-year period (2000 and 2012). *Design and Methods*. In a household survey conducted in 2012, in Dashtkhak/Kerman, 1200 individuals above 12 years of age completed a questionnaire to determine their frequency of substance abuse. The questionnaire included the following three areas: demographic characteristics, frequency of substance abuse and ease of access to various drugs. *Results*. Among 900 completed questionnaires, majority of the participants (61.8%) were below 30 years of age and among them 54.4% were male. Cigarette (17.0%), opium (15.7%) and opium residue (9.0%) were the most frequent substances abused on a daily basis. Based on the participant's opinion, we conclude that the ease of access to cigarette, waterpipe and opium contributed to their increase in consumption compared with earlier years. *Discussion and Conclusion*. The steady rise in substance abuse in rural communities demands immediate attention and emergency preventive measures from policy makers.

## 1. Introduction

Substance abuse poses a major political, social and health challenge worldwide [1]. Besides being a personal risk, addiction is a social problem and imposes harmful and permanent effects on society. At present, 3.6%–6.9% of adults (15–64-years-old individuals) are under the influence of illicit substances [2]. According to the World Drug Report 2013, since 2008, the number of illicit drug users has shown 18% increase; rise in population and ease of availability appears to be the two common reasons [2].

Substance abuse in Iran, one of the Middle East countries, has a historical origin. During World War I, among 250,000 individuals of the Tehran municipality, 25,000 were reported to be addicted to opium [3]. In recent centuries, opium has been prescribed for pain relief in Iran by traditional physicians. These traditions are stronger among rural populations owing to a lack of access to trained physicians. The usage of opium had been so common in rural areas that mothers often exposed babies to opium in an effort to calm them and sleep better [3]. The past two centuries have seen several changes with respect to handling illicit substances in Iran, from open access to death penalty for carrying illicit drugs. At present, Iran policy against illicit drugs is considered to be a combination of *war on drugs* and *harm reduction* strategies. With regards to opium use, Iran ranks among the top three countries in the world [4]. Therefore, epidemiological studies in Iran can provide better understanding for national policy makers as well as for other researchers in the world. Although several studies on the prevalence of substance abuse in Iran have been reported, the majority have been limited to urban areas [5, 6]; studies in rural areas were limited or often included small sample size [7]. Approximate one third of the Iran population lives in rural areas [8]; hence, it is critical to evaluate the substance abuse pattern in rural areas. In addition, the majority of epidemiological studies focussed on the trends of substance abuse because drug use behaviours are often dynamic, and with time are affected by various factors such as drug availability [9].

The present study aimed to investigate the trends of substance abuse among the rural communities of the Kerman province, the largest province in Iran, during a 12-year period by comparing data for the years 2000 and 2012. This region was selected for two reasons. First, Kerman as an eastern province, due to neighborhood with Afghanistan, the largest oipioid producing country in the world, is at higher risk of substance use, especially in youth group. A previous study reported that the usage of waterpipe, cigarette and alcohol, among high school boys, in this area was 51.5%, 34.6% and 7.27%, respectively [10]. Historically, Kerman had highest of opium usage in Iran [11]; second, accessibility of data from earlier years from this region [11] makes it easy to compare and analyse the changes with time. To the best of our knowledge, this is the first report describing the trend of drug abuse in rural Iran.

## 2. Methods

A household survey was conducted in 2012 in Dashtkhak, a northeastern village of Kerman province with a total population of 4416. The research was conducted under the approval of the Ethical Committee of Kerman University of Medical Sciences (Approval code: K/90/516). Following a brief meeting with the village council, details regarding the aim of the study, protocols and information regarding the questionnaire were explained to the participants. Confidentiality agreement and informed consent form were provided before the study. A researcher along with community health workers distributed the questionnaires to each house. One participant above 12 years of age per household was chosen to complete the questionnaire. Upon completion of the questionnaire, participants dropped them in a sealed ballot. The community health workers assisted the uneducated participants in the survey. Community health workers are employed by The Ministry of Health and provide primary health care in rural areas. Because of their acquaintance with the residents, their involvement in this research provided a great benefit to acquire data in a timely manner [12].

The validity and reliability of the questionnaire used in this study has been confirmed in previous studies [11]. It focussed primarily on three areas: The demographic features, details on substance abuse and the availability of substance. For details on substance abuse, substance abused throughout the lifetime, the last 30 days and every day were accounted [9]. Considering the pattern of substance abuse in Iran [5, 10], the questionnaire included the following substances: cigarette, waterpipe, opium, shireh (opium residues), heroin, cannabis, *shisheh*, alcohol and sedatives. Substance availability was counted using a four-degree Likert scale [9]. *Shireh* comprises opium residue obtained after opium consumption that is boiled and concentrated; this is more potent than opium. The primary component of shisheh is methamphetamine [13].

Data analysis was performed by calculating percentage values. The chi-square test was used to compare the frequency of substances abused between the two time points. Two separate multivariate logistic regression models for tobacco and the other drugs were included to assess the association between baseline characteristics and substance abuse. Data were analysed through the SPSS 20 software package.

## 3. Results

Among the 1200 distributed questionnaires, 900 participants responded and completed the questionnaires (response rate = 75.0%). Lack of interest and time proved to be the primary reasons for incomplete response. The average age and sex of non-respondents did not differ from those of respondents. The majority of respondents (61.8%) were below 30 years of age, and 54.4% among them were male (Table 1). Other demographic characteristics are presented in Table 1.

Opium, waterpipe and cigarette showed highest prevalence among all the other substance tested. Cigarettes, opium and *shireh* were on the most common daily abused drugs. Majority of the substances were consumed high among men compared with women (Table 2). Cigarette, opium and sedative usage was higher among the participants above 30 years compared with younger groups, whereas waterpipe consumption revealed a reverse pattern (Table 3). From the participant point of view, cigarette, opium and waterpipe were easily accessible compared with other substances (Table 4). Moreover, cigarette, opiates, heroin and sedatives consumption increased significantly in 2012 (Table 5).

Tobacco smoking was prevalent among male (OR = 10.8, CI 95%: 6.2–18.6) and unemployed participants (OR = 2.4, CI 95%: 1.4–4.3). Other drugs such as opium, heroin, cannabis, sedatives, alcohol and amphetamines were common among males (OR = 2.7, CI 95%: 1.9–4.0) and married (OR = 4.5, CI 95%: 2.4–8.5) and unemployed (OR = 2.4, CI 95%: 1.3–4.3) participants.

## 4. Discussion

Detailed analysis on the trend and pattern of substance abuse is necessary to develop preventive measures. The present study illustrates a substantial increase in substance abuse in a rural area over a 12-year period. Our study evaluates two different time periods in the same area using similar method, with a gap of 12 years; this offers the additional advantage for comparing the effect of substance abuse over time. Household surveys are considered as the gold standard for estimating the number of substance abusers [14], provided the participants are ensured of privacy and that they trust those collecting their information. Such mutual trust between village inhabitants and community health workers governs the rural regions in Iran [12]. The primary drawback of this study was its limitation to a single rural area, which requires caution in generalizing results. In addition, although response rates less than 70% were considered acceptable [15], non-participation of 25% of subjects in our study may limit data interpretation.

Kerman province, because of its borders with Afghanistan, is in the transit path for opium and heroin from Eastern borders to other parts of the country [5]. Since the early 19th century, heavy opium use among the natives has been connected to its easy access [3].

The ratio of male and female participants was similar in both 2000 and 2012. The participant population in the year 2000 was younger compared with that in 2012 (*P* < 0.05). This is probably because of an increase in average life span in Iran in recent years [8]. The lifetime substance abuse pattern showed a minor difference compared with that of the near-daily pattern. Opium, waterpipe and cigarette had highest prevalence of substance abuse throughout life and opium, *shireh* and cigarette among the daily abused substances (Table 2). This study reveals that opium abuse is endemic in this region [9] with lifetime prevalence higher than that of cigarette smoking (Table 2). In comparison with the data from 2000 showed that, in 2012, except for cannabis and alcohol, abuse of all other substances increased significantly. *Shireh* and heroin abuse enhanced 7-8 times. Based on anecdotal data, one of the reasons for this increase may be the rise in the cost of opium [16]. The consumption of substances, such as waterpipe and *shisheh*, was not investigated in 2000; these substance gained popularity during the last few years and are now considered as re-emerging drugs [11, 12, 17]. Although methamphetamine consumption was low compared with that of opiates, in urban areas, a gradual switch from traditional substances such as opium to synthetic substances such as methamphetamine is observed [17]. However, according to the current study, substance abuse patterns in rural areas differ from those in urban areas. Sedative consumption increased 6-fold, which is probably caused by easy availability of prescription drugs. An earlier study conducted in one of the rural regions of northern Iran reported that 13.5% of the participants abused opium almost daily and 28.1% smoked cigarette daily [7]. In another study, which analysed urine samples through anonymous unlinked testing, 14.5% showed opioid consumption, that is, similar to the rate of daily abuse of opium in our study (15.7%). A comparison of substance abuse among urban and rural population showed that opioid abuse is higher among rural population, whereas other substances such as alcohol waterpipe and cannabis were prevalent among urban population [10]. Substance consumption, except waterpipe, was higher among participants above 30 years of age compared with the younger age groups, indicating that waterpipe is regaining popularity among youth. In United States, it has been reported that except ecstasy and amphetamines, drug usage in the past year is relatively similar among adolescents in rural and urban regions [18]. Although new studies are in favour of higher usage of tobacco, cannabis and alcohol in U.S. and Australian rural students compared with urban students [19].

The substance abuse was also much higher among men compared with that among women (Table 3); the most obvious difference was observed for cigarette, suggesting the social stigma associated with cigarette smoking in women in Iranian culture [20], while the stigma associated with waterpipe consumption is significantly less [21]. Sedative consumption was similar among both men and women probably because of similar and easy access to prescription drugs and the prevalence of anxiety and insomnia in general population.

Access to *shisheh* was more difficult than that to other drugs. Approximately three quarters of rural population had easy access to cigarette and water pipe, and half of them had easy access to opium. Despite the law against usage of tobacco, sale of cigarette and waterpipe tobacco in public places is common in Iran. Waterpipe usage is common among youth and has increased in recent years, which can be often observed in coffee shops, parks and college dormitories [21]. Because alcohol was prohibited by religion, it was expected that access to alcohol be most difficult.

The logistic regression analysis showed that higher the difficulty towards access to tobacco, the less the probability of tobacco smoking (Table 6). In Iran, there is no close monitoring on tobacco sales to youth and cigarette smoking is socially well accepted in metropolitan areas than rural areas [20]. In rural areas, cultural barrier appears to play a role than geographical barrier, and the difficulty in access may be related to factors such as family supervision and cultural taboo on smoking by youth and women [21]. The higher probability of tobacco smoking among men was in-line with the earlier results from both the urban [20, 21] and rural [12] areas of Iran. The differences among men and women regarding drug abuse (OR = 2.7) were less prominent than tobacco smoking (OR = 10.8) (Table 6). It may be because of the fact that except for tobacco all other substance abuse is illegal in Iran [20]; while drug abuse is considered a taboo among men and women, usage of tobacco is socially accepted among men, whereas it is stigmatized among women [20, 21]. In contrast to drug abuse, age had no effect on tobacco smoking, perhaps because of the higher age at onset of drug abuse comparing with that of tobacco abuse [10, 21]. Drug abuse was more common among unemployed; a finding that may not imply causation because of an ambiguous order of variables (i.e. unemployment and drug abuse). Difficulty in access played a major role and clearly reduced drug abuse.

## 5. Conclusions

In conclusion, the present study shows increased prevalence of substance abuse among rural population in Iran. Easy access to the substance appears to contribute significantly to this trend. The pattern of drug usage among rural population differs from urban areas. The results of the present study emphasize the necessity of immediate multi-dimensional preventive measures to regulate substance abuse among rural communities.

## Figures and Tables

**Table 1:** Demographic characteristics of rural residents (*n* = 900).

Characteristic	Frequency	%
Age (yrs)		
12–19	140	15.6
20–29	221	24.6
30–39	194	21.6
40–49	147	16.3
50–59	98	10.9
≥60	100	11.1
Gender		
Male	490	54.4
Female	410	45.6
Education		
Illiterate	113	12.6
Primary school	207	23.0
Incomplete secondary	244	27.1
Complete secondary	219	24.3
College	117	13.0
Marital status		
Single	257	28.6
Married	643	71.4
Separated/divorced	0	0
Job		
Housewife	262	29.1
Employee	123	13.7
Worker	118	13.1
Student (school )	117	13.0
Student (college)	55	6.1
Farmer	50	5.6
Tradesman	24	2.7
Soldier	15	1.7
Jobless	68	7.6
Others	68	7.6

**Table 2:** Frequency of substance use among rural residents (*n* = 900).

Substance	Lifetime use			Past 30 days			Near-daily		
	*n*	%	95% CI	*n*	%	95% CI	*n*	%	95% CI
Cigarette	286	31.8	28.8–34.9	171	19.0	16.5–21.8	153	17.0	14.6–19.6
Waterpipe	321	35.7	32.6–38.8	99	11.0	9.1–13.2	33	3.7	2.6–5.0
Cannabis	18	2.0	1.2–3.9	8	0.9	0.4–1.7	5	0.6	0.2–1.2
Opium	333	37.0	33.9–40.2	180	20.0	17.5–23.7	141	15.7	13.4–18.2
Shireh (opium residue)	185	20.6	18.0–23.3	114	12.7	10.6–15.0	81	9.0	7.2–11.0
Heroin	39	4.3	3.1–5.8	25	2.8	1.8–4.0	22	2.4	1.6–3.6
Shisheh (amphetamine)	7	0.8	0.3–1.5	5	0.6	0.2–1.2	2	0.2	0.04–0.7
Alcohol	69	7.7	6.1–9.5	16	1.8	1.1–2.8	5	0.6	0.2–1.2
Sedatives	177	19.7	17.2–22.4	87	9.7	7.9–11.7	41	4.6	3.3–6.1

**Table 3:** Percentage of daily substance use among rural residents based on sex and age group (*n* = 900).

Substance	Sex			Age group (yrs)		
	Male	Female	*P* value	<30	≥30	*P* value
Cigarette	144 (29.4)	9 (2.2)	<0.001	26 (7.2)	127 (23.6)	<0.001
Waterpipe	26 (5.3)	7 (1.7)	0.004	23 (6.4)	10 (1.9)	<0.001
Cannabis	4 (0.8)	1 (0.2)	0.383	1 (0.3)	4 (0.7)	0.358
Opium	107 (21.8)	34 (8.3)	<0.001	17 (4.7)	124 (23.0)	<0.001
Shireh (opium residue)	68 (13.9)	13 (3.2)	<0.001	12 (3.3)	69 (12.8)	<0.001
Heroin	19 (3.9)	3 (0.7)	0.002	5 (1.4)	17 (3.2)	0.092
Shisheh (amphetamine)	1 (0.2)	1 (0.2)	1.00	0 (0.0)	2 (0.4)	0.247
Alcohol	3 (0.6)	2 (0.5)	0.255	3 (0.8)	2 (0.4)	0.363
Sedatives	23 (4.7)	18 (4.4)	0.828	10 (2.8)	31 (5.8)	0.036

**Table 4:** Perceived difficulty in getting each of drugs*.

Substance	Very easy	Easy	Difficult	Very difficult
Cigarette	474 (52.7)	232 (25.8)	63 (7.0)	131 (14.6)
Waterpipe	389 (43.2)	277 (30.8)	94 (10.4)	140 (15.6)
Cannabis	45 (5.0)	74 (8.2)	244 (27.1)	537 (59.7)
Opium	227 (25.2)	241 (26.8)	142 (15.8)	290 (32.2)
Shireh (opium residue)	188 (20.9)	213 (23.7)	160 (17.8)	339 (37.7)
Heroin	40 (4.4)	82 (9.1)	227 (25.2)	551 (61.2)
Shisheh (amphetamine)	14 (1.2)	41 (3.7)	209 (23.2)	636 (71.9)
Alcohol	50 (5.6)	92 (10.2)	211 (23.4)	547 (60.8)
Sedatives	136 (15.1)	171 (19.0)	176 (19.6)	417 (46.3)

*Figures are in percentages.

**Table 5:** Prevalence (%) of daily substance use among rural residents in 2000 and 2012.

Substance	2000 study *N* = 1668	Current study *N* = 900	*P* value
Cigarette	122 (7.3)	153 (17.0)	<0.001
Waterpipe	NS	33 (3.7)	—
Cannabis	6 (0.3)	5 (0.6)	0.683
Opium	91 (5.0)	141 (15.7)	<0.001
Shireh (opium residue)	22 (1.3)	81 (9.0)	<0.001
Heroin	5 (0.3)	22 (2.4)	<0.001
Shishehn (amphetamine)	NS	2 (0.2)	—
Alcohol	2 (0.1)	5 (0.6)	0.105
Sedatives	14 (0.8)	41 (4.6)	<0.001

*NS: not studied.

**Table 6:** Logistic regression analysis to examine baseline characteristics associated with tobacco and other drugs use.

Characteristics	Tobacco use			Other drugs use		
	Adjusted odds ratio	95% CI	*P* value	Adjusted odds ratio	95% CI	*P* value
Age						
<30 years	1	—	—	1	—	—
≥30 years	1.42	0.84–2.40	0.188	2.13	1.30–3.50	0.003
Sex						
Female	1	—	—	1	—	—
Male	10.78	6.24–18.60	0.001	2.74	1.87–3.99	0.001
Education						
Illiterate	1	—	—	1	—	—
Primary	1.52	0.43–1.44	0.193	0.94	0.55–1.62	0.836
Secondary	1.19	0.65–2.73	0.620	1.29	0.72–2.34	0.394
Higher	0.78	0.33–2.02	0.340	0.61	0.35–1.08	0.090
Marital status						
Unmarried	1	—	—	1	—	—
Married	1.78	1.01–3.20	0.049	4.46	2.35–8.46	0.001
Job						
Employed	1	—	—	1	—	—
Unemployed	2.42	1.36–4.28	0.003	2.38	1.31–4.34	0.005
Access difficulty	0.76	0.59–0.99	0.042	0.64	0.50–0.82	0.001
